# IGF1R and MAPK15 Emerge as Potential Targets of Pentabromobenzylisothioureas in Lung Neuroendocrine Neoplasms

**DOI:** 10.3390/ph13110354

**Published:** 2020-10-29

**Authors:** Ewelina Motylewska, Marcin Braun, Zygmunt Kazimierczuk, Hanna Ławnicka, Henryk Stępień

**Affiliations:** 1 Department of Immunoendocrinology, Chair of Endocrinology, Medical University of Lodz, Pomorska 251, 92-213 Lodz, Poland; ewelina.motylewska@umed.lodz.pl (E.M.); hanna.lawnicka@umed.lodz.pl (H.Ł.); 2 Department of Pathology, Chair of Oncology, Medical University of Lodz, Pomorska 251, 92-213 Lodz, Poland; braunmarcin@gmail.com; 3 Department of Chemistry, Warsaw University of Life Sciences, Nowoursynowska 159C, 02-787 Warsaw, Poland; zkazimierczuk@gmail.com

**Keywords:** bronchopulmonary neuroendocrine neoplasm, IGF1R, MAPK15, PKD1, ZKK, pentabromobenzylisothioureas, H727, small cell lung cancer, large cell neuroendocrine carcinoma, carcinoid

## Abstract

Pentabromobenzylisothioureas are antitumor agents with diverse properties, including the inhibition of MAPK15, IGF1R and PKD1 kinases. Their dysregulation has been implicated in the pathogenesis of several cancers, including bronchopulmonary neuroendocrine neoplasms (BP-NEN). The present study assesses the antitumor potential of ZKKs, a series of pentabromobenzylisothioureas, on the growth of the lung carcinoid H727 cell line. It also evaluates the expression of MAPK15, IGF1R and PKD1 kinases in different BP-NENs. The viability of the H727 cell line was assessed by colorimetric MTT (3-(4,5-dimethylthiazol-2-yl)-2,5-diphenyl-tetrazolium bromide) and its proliferation by BrdU (5-bromo-2′-deoxyuridine) assay. Tissue kinase expression was measured using TaqMan-based RT-PCR and immunohistochemistry. ZKKs (10^−4^ to 10^−5^ M) strongly inhibited H727 cell viability and proliferation and their antineoplastic effects correlated with their concentrations (*p* < 0.001). IGF1R and MAPK15 were expressed at high levels in all subtypes of BP-NENs. In addition, the SCLC (small cell lung carcinoma) patients demonstrated higher mRNA levels of IGF1R (*p* = 0.010) and MAPK15 (*p* = 0.040) than the other BP-NEN groups. BP-NENs were characterized by low PKD1 expression, and lung neuroendocrine cancers demonstrated lower PKD1 mRNA levels than carcinoids (*p* = 0.003). ZKKs may suppress BP-NEN growth by inhibiting protein kinase activity. Our results suggest also a possible link between high IGF1R and MAPK15 expression and the aggressive phenotype of BP-NEN tumors.

## 1. Introduction

Although neuroendocrine neoplasms (NENs) are rare malignancies, their incidence is increasing. BP-NENs (bronchopulmonary neuroendocrine neoplasm) exist as four separate entities: typical carcinoid (TC), atypical carcinoid (AC), small cell lung carcinoma (SCLC) and large cell neuroendocrine carcinoma (LCNEC) [1]. According to the new nomenclature, well-differentiated TC and AC are classified as neuroendocrine tumors (NETs) and poorly differentiated SCLC and LCNEC as neuroendocrine carcinomas (NECs) [2]. The different biological and clinical characteristics of lung NETs and NECs result in a need for distinct therapeutic approaches. While the main treatment option in lung NEC patients is chemotherapy [3,4], a range of options are available for advanced NETs, with everolimus (an mTOR inhibitor) being the only approved agent for targeted therapy [5,6,7].

The ZKKs ZKK1, ZKK2 and ZKK3 are a series of pentabromobenzylisothioureas, distinguished by the presence of additional substituents on their nitrogen atoms [8], which have been shown to suppress the growth of several neoplastic cell lines [8,9,10]. Substituted S-benzylisothioureas have been found to demonstrate various biological properties such as the inhibition of nitric oxide synthases (NOSs) [11,12] and indoleamine-2,3-dioxogenase (IDO) [13], which could contribute to an anticancer effect [14,15,16]. In addition, ZKK3 has also been demonstrated to act as a multi-kinase inhibitor, targeting the kinases implicated in carcinogenesis in different tumors, such as insulin-like growth factor 1 receptor (IGF1R), mitogen-activated protein kinase 15 (MAPK15) and protein kinase D1 (PKD1) [9].

IGF1R is a cell-surface heterotetrameric tyrosine kinase receptor with potent anti-apoptotic and pro-survival capacities. It plays a key role in carcinogenesis, indicated by the fact that various tumors demonstrate abundant IGF1R expression [17,18,19,20]. IGF1R is also considerably expressed in all four BP-NEN entities [21,22,23,24] and has been shown to interfere with the action of mTOR inhibitors [25,26], which makes it an interesting potential target for BP-NEN treatment. Indeed, some IGF1R inhibitors have undergone preliminary testing in patients with BP-NENs, both alone and in combination with other drugs [27,28].

The mitogen-activated protein kinases (MAPKs) are a superfamily of serine/threonine protein kinases [29]; of these, the most recently identified [30], and the least studied so far, is MAPK15, originally known as extracellular signal-regulated kinase 7/8 (ERK7/ERK8). MAPK15 is present in a number of tissues; however, its highest expression is observed in the lung and kidney [30]. It is still unclear whether MAPK15 acts as a protooncogene or a tumor suppressor [29]. Some evidence suggests that it plays a role in the carcinogenesis of male germ cell tumors [31], as well as colon [32] and gastric [33] cancers, while other studies indicate that it also maintains genome stability and suppresses tumorigenesis in mammary [34,35,36] and lung tissues [36].

PKD1 is a serine/threonine kinase belonging to the Ca++/Calmodulin-dependent kinase superfamily and is known to regulate several biological processes including cell proliferation, migration, invasion, apoptosis and angiogenesis [37]. The *PRKD1* gene generally exhibits a low frequency of somatic mutations in pan-cancer analysis [37,38]. However, PKD1 over- and underexpression has been observed in several cancer types, with the expression profile being specific to tumor tissue. Although PKD1 was found to induce mostly protumor effects, it has been demonstrated to have antiproliferative activities in lung, stomach and prostate cancers [38]. Interestingly, lung NET patients have demonstrated comparable levels of PKD1 mRNA in blood and tumor tissues [39]. PKD1 has been included in a blood-based set of 51 NET-specific transcripts, which could be useful for diagnosis and treatment monitoring in bronchopulmonary and gastroenteropancreatic NET patients [39,40].

The aim of this study was to investigate the effect of ZKKs on the growth of BP-NEN in vitro and to assess the abundance of IGF1R, MAPK15 and PKD1 in different BP-NEN subtypes.

## 2. Results

### 2.1. H727 Carcinoid Cell Line

**Figure 1:**
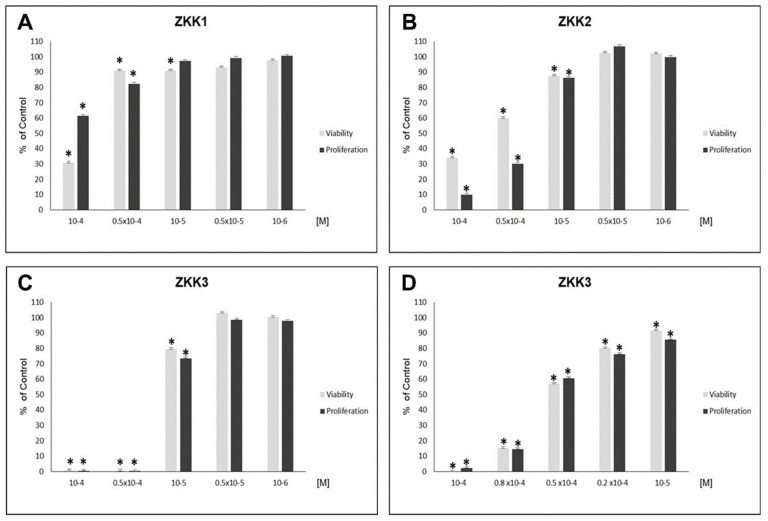
The effect of ZKK1-3 on the growth of H727 bronchial carcinoid cell line assessed by Mosmann and BrDU (5-bromo-2′-deoxyuridine) incorporation methods in 72-h culture. The data are presented as mean ± SEM and are for seven biological replicates, * *p* < 0.05 vs. corresponding control group by one-way ANOVA with LSD test (the least significant difference). The figure represents one of two independent experiments. (**A**)—the effect of ZKK1; (**B**)—the effect of ZKK2; (**C**,**D**)—the effect of ZKK3.

ZKK1 inhibited H727 growth in the MTT (3-(4,5-dimethylthiazol-2-yl)-2,5-diphenyl-tetrazolium bromide) method (to 30–91% of control values) at concentrations of 10^−4^, 0.5 × 10^−4^ and 10^−5^ M, and in the BrdU (5-bromo-2′-deoxyuridine) method (to 61–92% of control values) at concentrations of 10^−4^ and 0.5 × 10^−4^ M (Figure 1A).

ZKK2 inhibited cell viability to 34–88% of control values and reduced BrdU incorporation in H727 cells to 10–86% of control values at the highest concentrations (10^−4^, 0.5 × 10^−4^, 10^−5^ M) (Figure 1B).

At the highest concentrations (10^−4^, 0.5 × 10^−4^, 10^−5^ M) ZKK3 intensively inhibited H727 growth, i.e., to 1–80% of control values in the MTT method, and to 0.5–73% of control values in the BrdU method (Figure 1C). Therefore, further experiments with ZKK3 at additional intermediate concentrations, viz. 10^−4^, 0.8 × 10^−4^, 0.5 × 10^−4^, 0.2 × 10^−4^ and 10^−5^ M, were performed. At all studied concentrations, ZKK3 decreased H727 viability to 0, 15, 57, 80 and 92% of control values, respectively, and reduced proliferation to 2, 15, 61, 76 and 86% of control values, respectively (Figure 1D).

All studied substances demonstrated a correlation between concentration and intensity of H727 cell growth inhibition (Table 1).

### 2.2. Kinase Inhibition Profile of ZKKs

**Figure 2:**
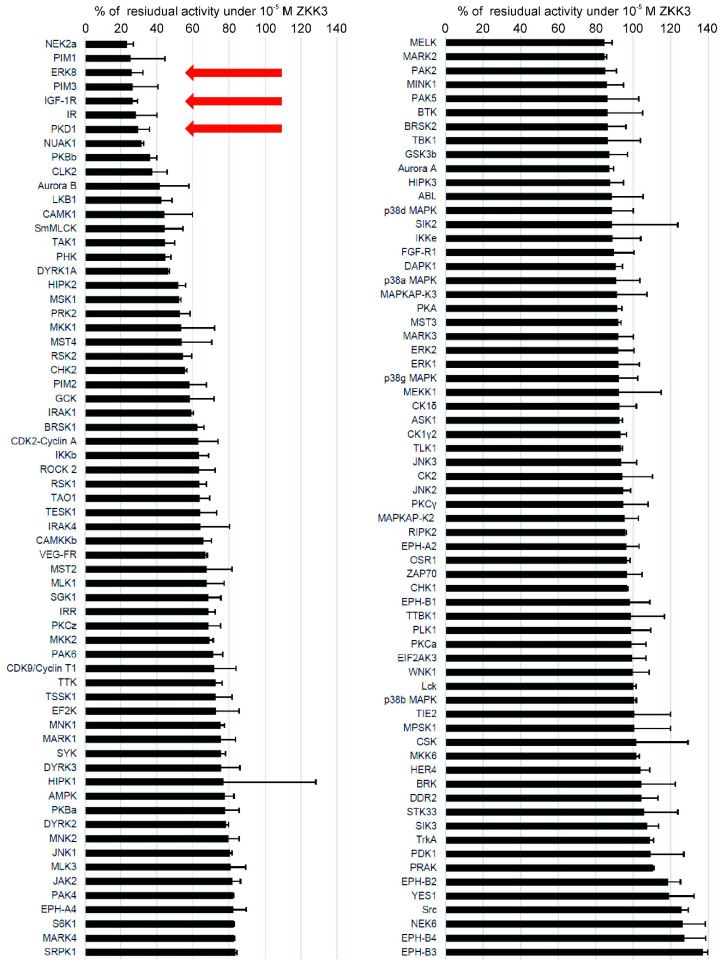
The selectivity profile of ZKK3 (10^−5^ M) determined against a large kinase panel [9]. Residual activity of kinases is shown as percentage of the control without inhibitor. X ± SD.

The structurally related ZKKs exhibited multi-kinase inhibitor activity, targeting several kinases known to play key roles in the metabolism of normal and malignant cells, i.e., IGF-1R, ERK8 and PKD1 [9] (Figure 2).

### 2.3. MRNA Levels of IGF1R, MAPK15 and PKD1 in BP-NEN Patients’ Tumors

Forty-five patients with good quality total RNA were chosen for analysis. In the whole BP-NEN group, the highest mRNA expression was demonstrated by IGF1R (RQ 6.66 (3.92–11.92)), which was more plentiful than MAPK15 mRNA (RQ 4.77 (2.48–12.32)). PKD1 demonstrated the lowest mRNA expression in BP-NENs (RQ 2.03 (0.95–3.09)).

**Figure 3:**
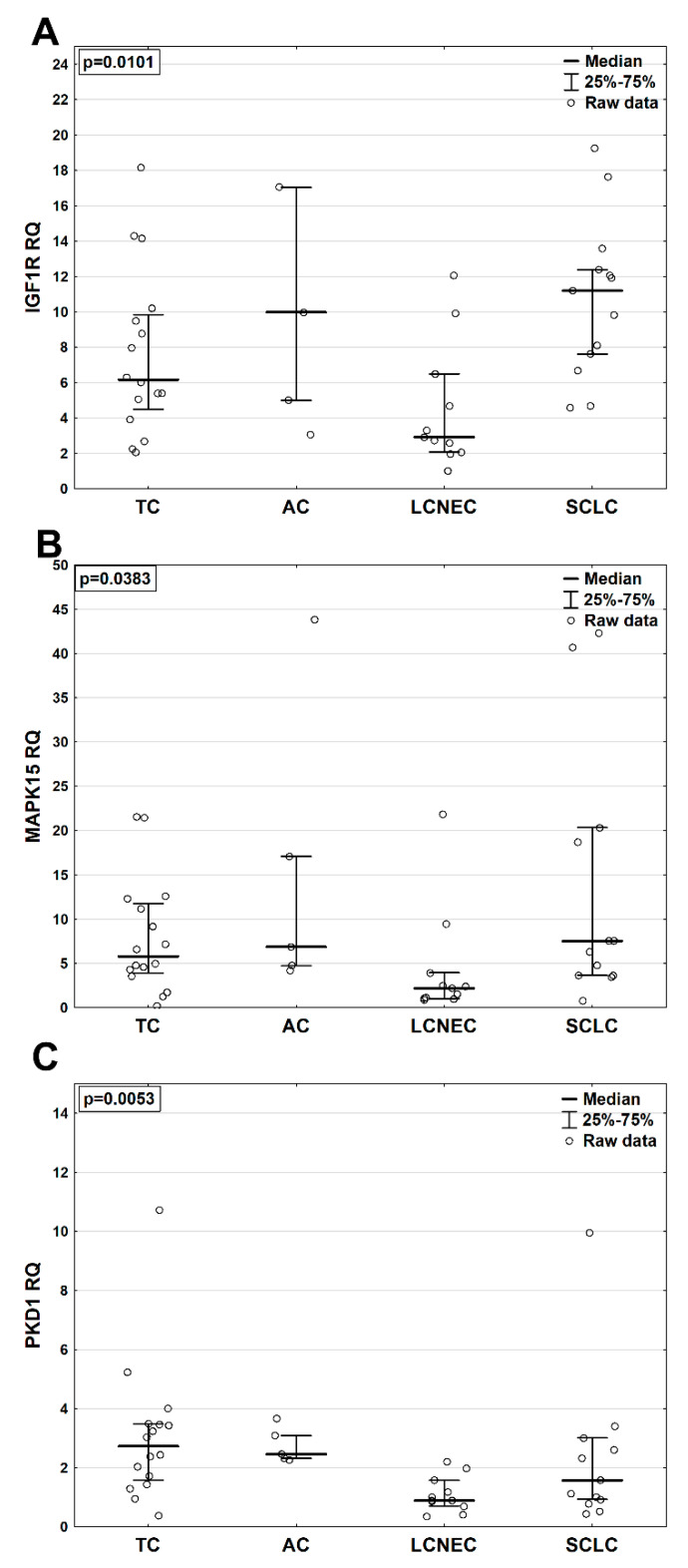
mRNA expression of kinases in different BP-NEN (bronchopulmonary neuroendocrine neoplasm) entities: (**A**)—IGF1R expression, (**B**)—MAPK15 expression, (**C**)—PKD1 expression. TC—typical carcinoid; AC—atypical carcinoid; SCLC—small cell lung carcinoma; LCNEC—large cell neuroendocrine carcinoma. *p* < 0.05 by ANOVA Kruskall–Wallis.

IGF1R mRNA levels were significantly higher (*p* = 0.01) in SCLC (RQ 11.19 (7.60–12.38)) than in other entities (RQ 2.92 (2.07–6.49); 9.97 (4.98–17.03); 6.16 (4.49–9.84) for LCNEC, AC and TC, respectively) (Figure 3A). Similarly, MAPK15 mRNA expression was also elevated (*p* = 0.04) in SCLC (RQ 7.50 (3.63–20.31)) compared to other BP-NENs (RQ 2.19 (1.02–3.94); 6.85 (4.72–17.06); 5.78 (3.91–11.73) for LCNEC, AC and TC, respectively) (Figure 3B). PKD1 mRNA expression was significantly increased in NETs compared to NECs (*p* = 0.003) (data not shown), and the highest levels were detected in TC (RQ 2.72 (1.57–3.47)) vs. SCLC (RQ 1.57 (0.93–3.01)), LCNEC (RQ 0.88 (0.70–1.57)) and AC (RQ 2.46 (2.32–3.09)) (*p* = 0.005) (Figure 3C). In addition, IGF1R mRNA expression positively correlated with MAPK15 (*p* < 0.05; R = 0.47) and PKD1 (*p* < 0.05; R = 0.51) mRNA levels in the BP-NEN samples.

### 2.4. Protein Levels of IGF1R, MAPK15 and PKD1 in BP-NEN Tumors

**Figure 4:**
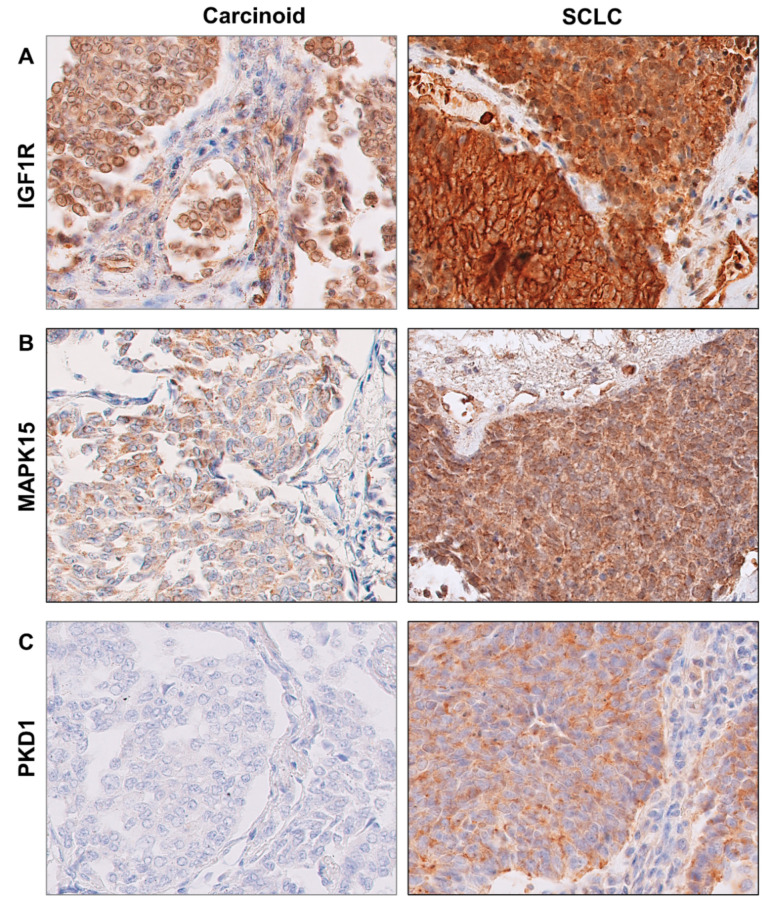
Examples of representative positive immunohistochemical staining for kinases in well-differentiated and poorly differentiated BP-NENs (bronchopulmonary neuroendocrine neoplasms). TC—typical carcinoid; SCLC—small cell lung carcinoma. (**A**)—IGF1R expression, (**B**)—MAPK15 expression, (**C**)—PKD1 expression.

In BP-NEN patients, the most frequent immunoreactivity was observed for IGF1R kinase, being present in 53 (90%) samples (Figure 4A). Positive immunoreactivity for MAPK15 was detected in 49 (83%) BP-NEN sections (Figure 4B). PKD1 kinase demonstrated very weak immunoreactivity in BP-NEN specimens (Figure 4C). PKD1 protein expression was observed in only 15 (25.0%) sections, which exhibited only weak staining (IRS 1).

No statistically significant differences in IGF1R, MAPK15 and PKD1 protein levels were observed between different BP-NEN entities. However, IGF1R protein tended to be particularly abundant in SCLC, as it was found to be present in 23 (96%) specimens, of which 11 (46%) showed the most intensive staining (IRS 3). IGF1R immunoreactivity was also detected in the TC group, in which 10 (62.5%) samples were characterized by IRS 2. Most MAPK15 protein expression in BP-NENs had an intensity of IRS 1, which was observed in nine (56%) TC, four (66.7%) AC, nine (37.5%) SCLC and six (46%) LCNEC. However, MAPK15 protein expression tended to be greater in the SCLC sections: only in this group, 13 samples (56%) were found to demonstrate IRS2 and IRS3.

No significant associations were found between mRNA and protein levels for the studied kinases.

### 2.5. Survival Analyses

A total of 23 deaths occurred within the BP-NEN group; median overall survival (OS, (IQR)) was 1.4 years (0.1–11.00). The median OS within studied subgroups was 2.4 years (1.6–2.7) for TC, 4.2 years (3.0–9.7) for AC, 2.1 years (0.9–2.6) for LCNEC and 0.7 years (0.3–1.2) for SCLC (Table 1). The differences in OS between BP-NEN entities were significant (*p* = 0.0002). No patients in the TC or AC groups died. Only borderline significant differences in OS were observed between LCNEC and SCLC groups (*p* = 0.054). Further statistical analysis of survival was performed only in the lung NEC subgroup and revealed that patients with positive PKD1 protein expression demonstrated shorter OS than those with no PKD1 expression (HR 15.09 (1.68–135.94), *p* = 0.016). In turn, the lung NET subgroup was further examined on the basis of recurrence data. Tumor relapses were reported in the TC group (one case) and in the AC group (two cases); however, due to the small number of events, they could not be analyzed statistically.

## 3. Discussion

The present study shows that ZKK1, ZKK2 and ZKK3 may be effective against BP-NEN in vitro. This is the first study to assess mRNA and protein expression of IGF1R, MAPK15 and PKD1 kinases in all four entities of BP-NENs. Our findings indicate that IGF1R and MAPK15 were abundantly expressed in BP-NEN tissues and that they were present at higher levels in SCLC than in other entities.

ZKKs are compounds with diversified properties, including inhibition of IGF1R, MAPK15 and PKD1 kinases [9]. ZKKs have demonstrated antineoplastic potential against various malignancies; for example, ZKK1-3 (1 × 10^−5^ − 5 × 10^−5^ M) have been found to inhibit the growth of human leukemia, glioblastoma and prostate cancer cell lines in a time- and concentration-dependent manner [8,9,10]. Structural modifications of ZKK1 influence its efficacy, with N-substitution with short alkyl groups increasing cytotoxic activity [10], as demonstrated by ZKK2 and ZKK3 [8,9,10]. Similarly, in the present study, the most potent cell growth suppression was induced by ZKK3, which completely arrested H727 carcinoid growth at its highest concentration (10^−4^ M). The H727 cell line has been previously reported to express IGF1R and PKD1 kinases [24,39], which suggests that ZKKs may suppress growth by inhibiting these kinases. In order to explore this further in a broader context, the study assessed the expression of kinases targeted by ZKKs in different BP-NEN tissues.

Several immunohistochemical studies revealed the presence of IGF1R protein in SCLC tissues [21,41,42,43]. These papers indicated similar percentages of positive immunostaining for IGF1R in SCLC specimens, ranging from 71 to 81% of SCLC samples, with intensity varying from weak to moderate. In the present study, positive immunoreactivity for IGF1R was observed in 96% SCLC tissues, most of which demonstrated strong staining intensity. Similarly, no previous studies have found IGF1R expression to have any prognostic value in SCLC patients [42,43], except in one cohort with extensive SCLC, in which IGF1R expression was found to be associated with longer OS [42]. Similarly to our present findings, IGF1R expression has previously been demonstrated in other BP-NENs, with no difference in IGF1R level observed between SCLC and LCNEC [21] and between TC and AC tissues [22,23,24,44]. However, our work is the first to offer a complete comparative analysis of IGF1R expression between all four BP-NEN entities and suggest a link between higher IGF1R levels and the aggressive phenotype of tumors. This is an important finding, as in vitro and in vivo studies have found IGF1R overexpression to promote the growth of SCLC [41,45] by interacting with the apoptosis-related Bcl-2 protein family members and AKT pathway [41].

The role of MAPK15 in carcinogenesis, including lung neoplasms, remains relatively understudied and ambiguous. RT-PCR and Western blot analyses have revealed relatively high MAPK15 expression in a few non-small cell lung cancer (NSCLC) lines [46]; however, MAPK15 levels were found to be clearly detectable in lung biopsies of normal tissues but markedly lower in lung carcinomas, including one case of SCLC [36]. MAPK15 expression was also detected by immunohistochemical staining in an atypical carcinoid [47]. Our present findings indicate that MAPK15 is moderately expressed in all types of BP-NEN, with higher levels in SCLC tissues; however, as it is the activation of the signaling pathway that is a fundamental requirement in transformation, not the overexpression itself, the clinical relevance of our findings remains unclear.

Similarly, the impact of PKD1 in lung neoplasms is unclear due to the paucity of studies and often contradictory results. PKD1 has been shown to be down- [48] or upregulated [38] in NSCLC. It has been proposed that cooperation of PKD1 with E-cadherin or the mTORC1/S6K1 (mammalian target of rapamycin complex 1/p70 ribosomal protein S6 kinase 1) pathway has an antitumor effect in NSCLC [48,49], while PKD1 activation through a PKC-dependent pathway has been shown to mediate pro-tumor responses in SCLC cells [50]. Our study found PKD1 mRNA levels to be higher in lung NETs than in lung NECs. Similarly, the PKD1 mRNA expression has been found to be characteristic for carcinoids and included in the NET-specific transcript set, which could be useful in the diagnostic and monitoring procedures [39]. However, in our tumor samples, PKD1 protein was not expressed, or expressed at low levels. Intriguingly, positive PKD1 protein expression was associated with worsened OS in lung NEC patients.

Interestingly, the statistical analysis did not reveal any significant relationships between the mRNA and protein levels of particular kinases. However, a correlation between increased mRNA and protein expression in SCLC was observed for IGF1R and MAPK15. Importantly, such disassociations between the levels of mRNA and the protein product are commonly observed and connected with post-transcriptional, translational and protein degradation regulation [51,52].

Discussing the limitations of our pilot study, it is important to refer to the relatively small size of the study cohort. This was necessitated by the very low incidence of BP-NENs, especially lung NETs, and the fact that the study was restricted to a single center. In addition, it is difficult to obtain adequate primary SCLC samples for expression studies, as surgery is not a standard therapeutic option for this entity. However, our results of survival analysis are in line with global survival data in BP-NEN patients, indicating that despite its limited size, the cohort is representative for the general population. Moreover, the use of only one cell line can be perceived as a limitation of our study. However, it is important to emphasize that there are only two commercially available cell lines representing lung typical carcinoid, from which the H727 line is the most popular. The fact of limited pulmonary carcinoid cell line resources hinders efforts to understand the biology of this rare type of lung tumor and develop new drugs. In this context, we believe that our results are of value to be reported.

Our results may be important in the therapeutic context. Although targeting the IGF axis in different malignancies has yielded disappointing results in phase III trials [20] and research on MAPK15 targeting drugs is at an very early stage [29], these strategies are still regarded as promising alternatives in precision oncology. Our findings in the tumoral tissues, as well as the in vitro experiments with ZKK multi-kinase inhibitors, suggest that inhibition of IGF1R and MAPK15 could also be potentially effective in lung NET treatment.

## 4. Materials and Methods

### 4.1. Cell Line and Culture Condition

The human lung carcinoid cell line H727 obtained from the American Type Culture Collection (ATCC; CRL-5815™) was used. The cells were cultured in RPMI medium (ATCC; Cat No 30–2001) containing 10% fetal bovine serum (Biochrom; Berlin, Germany; Cat No S0115) and 100 U/mL penicillin and 100 μg/mL streptomycin solution (Sigma; Saint Louis, USA; Cat. No P0781). The cells were passaged every 7 days with 0.05% trypsin/0.02% EDTA (Trypsin-EDTA, Sigma; Saint Louis, USA; Cat. No T3924). The cells were regularly tested for mycoplasma contamination (MycoProbe Mycoplasma Detection Kit, R&D; Minneapolis, USA; Cat. No CUL001B).

### 4.2. Substances

A series of new pentabromobenzylisothioureas carrying additional substituents on nitrogen atoms were tested: ZKK1 (S-(2,3,4,5,6-pentabromobenzyl)isothiouronium bromide); ZKK2 (N-Methyl-S-(2,3,4,5,6-pentabromobenzyl)isothiouronium bromide) and ZKK3 (N,N′-Dimethyl-S-(2,3,4,5,6-pentabromobenzyl)isothiouronium bromide). ZKK1, ZKK2 and ZKK3 were synthesized as described previously [8].

ZKK1, ZKK2 and ZKK3 were dissolved in 96% ethanol and serum-free culture medium and added to the appropriate wells at final concentrations of 10^−4^ − 10^−6^ M (the highest concentration of ethanol was 1.5% (vol) in the 10^−4^ M wells). Three control groups (corresponding to 10^–4^,10^–5^ or 10^–6^ M ZKK groups) with different ethanol concentrations were used in every experiment. For this purpose, serum-free culture medium and 96% ethanol at the same concentrations as the solvent in corresponding ZKK group were added to the control wells. Each experiment was repeated independently at least twice, with seven biological replicates per group.

### 4.3. Inhibition of Protein Kinases by ZKKs

Kinase profiling for the series of structurally similar ZKKs (i.e., ZKK1-3) was performed with the example of ZKK3. The inhibitory activity of 10^−5^ M ZKK3 was tested against a panel of 130 selected protein kinases; the compound was screened in duplicate. The analysis was performed in the International Centre for Kinase Profiling (the University of Dundee, Scotland).

### 4.4. Cell Viability Study (Mosmann Method)

The MTT (3-(4,5-dimethylthiazol-2-yl)-2,5-diphenyl-tetrazolium bromide) colorimetric assay was used as an indicator of cell viability/cytotoxicity (EZ4U kit, Cell Proliferation & Cytotoxity Assay, Biomedica; Wien, Austria; Cat. No BI-5000). The MTT analysis on H727 cells was performed as previously reported [53], but with a density of 1.5 × 10^4^ cells per well. The optical density (OD) of each sample was measured by a microplate reader at 450 nm. The results of the MMT analysis were presented as percentage of OD in the corresponding control group.

### 4.5. Cell Proliferation Study

H727 cell proliferation was quantified using colorimetric immunoassay based on the measurement of 5-bromo-2′-deoxyuridine (BrdU) incorporation during DNA synthesis (Cell proliferation ELISA, BrdU, Roche; Penzberg, Germany; Cat. No 11 647 229 001). H727 cell proliferation analysis was performed as previously reported [53]; however, a density of 1.5 × 10^4^ cells per well was used. The optical density (OD) of each sample was measured by a microplate reader at 450 nm. The results were presented as percentage of OD in the corresponding control group.

### 4.6. Study Cohort

The study group comprised 49 patients (27 males, 22 females) with median age of 65 years (60.00–70.00) (Table 2). Samples were provided as 60 formalin-fixed, paraffin-embedded tumor blocks (FFPEs) from the Medical University of Lodz, Poland. All tumors were classified according to the WHO 2004 or 2015 classification of lung neoplasms [54,55]. The study was approved by the Institutional Board of the Medical University of Lodz (Number RNN/145/18/KE).

All participants had been recently diagnosed with BP-NENs (2008 to 2019): 11 patients were diagnosed with TC, 5 with AC, 22 with SCLC and 11 with LCNEC. As no significant differences were found between the patients with regard to valid clinical parameters, all patients were further divided into two groups with NETs (*n* = 16) and NECs (*n* = 33) for statistical analysis.

Individual clinical data of patients are available in Supplementary Materials (Table S1).

### 4.7. IGF1R, MAPK15 and PKD1 MRNA Expression

FFPEs were used for mRNA analysis. The total RNA isolation and cDNA generation was performed as described previously [56]. Standard TaqMan^®^ Gene Expression Assays (ThermoFisher Scientific, Pleasanton, CA; Cat. No 4331182) were used to measure mRNA expression: mitogen-activated protein kinase 15 (MAPK15, Hs00933427_g1), insulin like growth factor 1 receptor (IGF1R, Hs00609566_m1), polycystin 1, transient receptor potential channel interacting (PKD1, Hs00947377_m1) and actin beta (ACTB, Hs 01060665_g1) as the endogenous control. TaqMan PCR assays were performed as previously reported [56]. All reactions were run in duplicate. Kinase expression levels were calculated using RQ (the 2^–ΔΔCt method).

### 4.8. Immunohistochemistry for IGF1R, MAPK15 and PKD1 Protein Expression

Immunohistochemical protein expression was studied in FFPE samples using Mouse Monoclonal (7e12) Anti-Polycystin 1/PC1 Antibody (Abcam; Cambridge, UK; Cat. No AB74115), Rabbit Polyclonal MAPK15 Antibody (ElabScience; Houston, TX, USA; Cat. No E-AB-40233) and Rabbit Policlonal Anti-IGF1R Antibody (Sigma-Aldrich; Saint Louis, MI, USA, Cat. No AB-1161). The following positive controls were set up: MAPK15 and PKD1 on renal carcinoma, and IGF1R on breast cancer. All immunohistochemical procedures were performed as previously reported [56]. Cytoplasmic staining was considered for the evaluation of MAPK15 expression, cytoplasmic and nuclear for IGF1R expression and cytoplasmic and membranous for PKD1 expression. Negative MAPK15, IGF1R and PKD1 staining in tumor sections was defined as IRS 0; positive samples were evaluated as IRS 1, 2 or 3 according to staining intensity. Following this, IRS 0 and 1 were combined into one group and IRS 2 and 3 into a second for statistical analysis.

The stained sections were examined by a BX43 Light Microscope (OLYMPUS Europa SE; CO, Hamburg, Germany). Images were recorded using an UltraFast Scanner (Philips IntelliSite Solution, Best, The Netherlands) with DigiPath™ Professional Production Software (Xerox, Norwalk, CT, USA).

### 4.9. Statistical Analysis

The normality of distribution of continuous variables was determined using the Shapiro–Wilk test. Normally distributed variables were compared using the Student’s t-test or one-way analysis of variance (ANOVA) and non-normally distributed variables with the Mann–Whitney U-test (or ANOVA Kruskall–Wallis). Categorical variables were tested using the χ2, two-tailed Fisher’s or Yates exact test. Bonferroni’s correction was used for multiple comparison. The degree of correlation was tested using Spearman’s rank or Pearson coefficient. For the outcome analyses, overall survival (OS) was defined as the time period from diagnosis to last follow-up, with censoring of live patients at the last follow-up. OS data are presented as Kaplan–Meier survival curves. The OS values within subgroups were compared with the log-rank test. *P*-values < 0.05 were considered statistically significant. Statistical analysis was carried out using the Statistica 13.1 PL package (StatSoft, Tulsa, OK, USA).

## 5. Conclusions

Our findings indicate that ZKKs effectively inhibit the growth of bronchial neuroendocrine neoplasms in vitro and that BP-NEN are characterized by abundant expression of the ZKK targets IGF1R and MAPK15. These preliminary but complementing results legitimize further functional research in the field of MAPK15 and IGF1R as potential targets in BP-NEN.

## Figures and Tables

**Table 1:** Correlations between the ZKK concentration [M] and H727 cell growth inhibition (residual growth expressed as % of control group). MTT-3-(4,5-dimethylthiazol-2-yl)-2,5-diphenyl-tetrazolium bromide; BrdU-5-bromo-2′-deoxyuridine; R—Pearson’s correlation coefficient.

	MTT Method		BrdU Method	
	*p*	R	*p*	R
ZKK1 (10^–4^ to 10^–6^)	<0.001	0.679	<0.001	0.859
ZKK2 (10^–4^ to 10^–6^)	<0.001	0.903	<0.001	0.908
ZKK3 (10^–4^ to 10^–6^)	<0.001	0.912	<0.001	0.925
ZKK3 (10^–4^ to 10^–5^)	<0.001	0.957	<0.001	0.936

**Table 2:** Study cohort characteristics. TC—typical carcinoid; AC—atypical carcinoid; LCNEC—large cell neuroendocrine carcinoma; SCLC—small cell lung carcinoma; IHC—immunohistochemistry; RT-PCR—real-time PCR.

Variable	Number (%) or Median (IQR)				
	Overall	TC	AC	LCNEC	SCLC
Sex: male/female	27 (55.1)/22 (44.9)	6 (12.2)/5 (10.2)	2 (4.1)/3 (6.1)	7 (14.3)/4 (8.2)	12 (24.5)/10 (20.4)
Age at diagnosis (years)	65.0 (60.0–70.0)	63.4 (51.7–65.7)	60.0 (57.8–70.0)	64.2 (57.5–72.0)	67.2 (64.7–70.1)
Overall survival (years)	1.4 (0.1–11.0)	2.4 (1.6–2.7)	4.2 (3.0–9.7)	2.1 (0.9–2.6)	0.7 (0.3–1.2)
IHC/RT PCR samples	59 (100.0)/45 (100.0)	16 (27.1)/16 (35.6)	6 (10.2)/5 (11.1)	13 (22.0)/11 (24.4)	24 (40.7)/13 (28.9)

## Supplementary Materials

The following are available online at https://www.mdpi.com/1424-8247/13/11/354/s1, Table S1: Individual clinical data of patients.
